# Analysis of Moisture Content in Beetroot using Fourier Transform Infrared Spectroscopy and by Principal Component Analysis

**DOI:** 10.1038/s41598-018-26243-5

**Published:** 2018-05-22

**Authors:** Noel Nesakumar, Chanthini Baskar, Srinivasan Kesavan, John Bosco Balaguru Rayappan, Subbiah Alwarappan

**Affiliations:** 1 Electrodics and Electrocatalysis Division, CSIR-Central Electrochemical Research Institute, Karaikudi, 630003 Tamil Nadu India; 2 School of Computing, SASTRA Deemed University, Thanjavur, 613401 Tamil Nadu India; 3 School of Electrical and Electronics Engineering, SASTRA Deemed University, Thanjavur, 613401 Tamil Nadu India

## Abstract

The moisture content of beetroot varies during long-term cold storage. In this work, we propose a strategy to identify the moisture content and age of beetroot using principal component analysis coupled Fourier transform infrared spectroscopy (FTIR). Frequent FTIR measurements were recorded directly from the beetroot sample surface over a period of 34 days for analysing its moisture content employing attenuated total reflectance in the spectral ranges of 2614–4000 and 1465–1853 cm^−1^ with a spectral resolution of 8 cm^−1^. In order to estimate the transmittance peak height (*T*_*p*_) and area under the transmittance curve $({\int }_{{\bar{\nu }}_{i}}^{{\bar{\nu }}_{f}}{T}_{p}d\bar{\nu })$ over the spectral ranges of 2614–4000 and 1465–1853 cm^−1^, Gaussian curve fitting algorithm was performed on FTIR data. Principal component and nonlinear regression analyses were utilized for FTIR data analysis. Score plot over the ranges of 2614–4000 and 1465–1853 cm^−1^ allowed beetroot quality discrimination. Beetroot quality predictive models were developed by employing biphasic dose response function. Validation experiment results confirmed that the accuracy of the beetroot quality predictive model reached 97.5%. This research work proves that FTIR spectroscopy in combination with principal component analysis and beetroot quality predictive models could serve as an effective tool for discriminating moisture content in fresh, half and completely spoiled stages of beetroot samples and for providing status alerts.

## Introduction

Beetroot (*Beta vulgaris L*.) is cultivated throughout the world for its vegetable and juice value^1^. It contains high concentration of betaine, vitamin A, B6 and C, folic acid, protein, carbohydrates, potassium, iron, soluble fibre, sodium and magnesium^1,2^. It has attracted much attention not only because of its rich nutrient content but also because of its medicinal significance^3^. It also helps to minimize blood pressure, manages cardiovascular health, improves stamina and muscle power, maintains blood circulation and slows the progression of dementia^4–7^. According to a report by British Dietetic Association (BDA), beetroot contains anthocyanins which can minimize the effects of pollution on the body. In addition, it is used as a source of natural antioxidants which aids to protect cells against oxidative stress in humans^8,9^. Its medicinal values have been associated with the number and amount of nutrients present in it^4–7^. The amount of nutrients starts to break down after harvest. However, the rate of nutrient losses can be minimized by proper storage^10^.

During storage, it releases heat from respiration and subsequently loses moisture^11^. As a result, swift softening and decay progress which decreases the shelf-life and nutritional quality^11^. In order to preserve and to increase its storage stability, numerous preservation methods have been employed^3,12^. However, every preservation process decreases the amount of nutrients in it^3,12^. Especially, processes that expose beetroot to high levels of oxygen, light and heat cause the greatest nutrient loss. Salting, pickling, fermenting, drying, canning, freezing, pressure canning and dry salting are the commonly used preservation methods^13–16^. Of all these methods, freezing appears to be promising in maintaining its initial quality for few days. In order to retain the amount of nutrients, beetroots are stored in the refrigerator at a temperature of 0–4 °C^17^. This low temperature is sufficient to decrease the rate of microbial growth, deterioration and biochemical reactions.

Controlled cold storage plays a major role in extending shelf-life. Beets with the greens can be stored in the refrigerator for few days^17^. However, beets with the greens detached can be stored for 14 days in the refrigerator^17^. Storage reduces the moisture loss and increases shelf-life. Furthermore, its shelf-life can be extended by integrating refrigeration with a controlled atmosphere comprising of a mixture of carbon dioxide and oxygen in an airtight room^13–16^.

During fresh harvest, there are heterogenous shades of red and purple. However, during storage, the heterogeneity turns to dark shades of red and purple^3^. Visual quality loss (sprouting, dehydration, wrinkled, dull in vibrant red-purple colour) takes place if no proper refrigerated storage is used, limiting its commercial value^3^. The retention of vibrant red-purple colour during refrigerated storage is usually considered as a measure of quality^18,19^. But, together with slight colour (vibrant red-purple) change, there can be aroma changes which could be adjudicated negatively by consumers and producers. Due to these reasons, developing new analytical methods for monitoring the moisture level of beetroot have recently gained attention^13–16^. To date, numerous methods have been proposed to measure and to detect the moisture level of beetroot^13–16^. However, these analytical methods require sample pre-treatment. On the other hand, Fourier transform infrared spectroscopy (FTIR) doesn’t require sample pretreatment^19^. To the best of our knowledge, there are no reports in literature employing FTIR for the estimation of its moisture level. Thus, the main objective of the present research was to examine the impact of decrease of moisture content on its quality using FTIR spectroscopy. Also, principal component analysis (PCA) and Gaussian curve fitting algorithm were applied to FTIR data to develop quality predictive models for the determination of its moisture level during cold storage.

## Results and Discussion

### Characterization of FTIR spectra of fresh and spoiled beetroot samples

**Figure 1:**
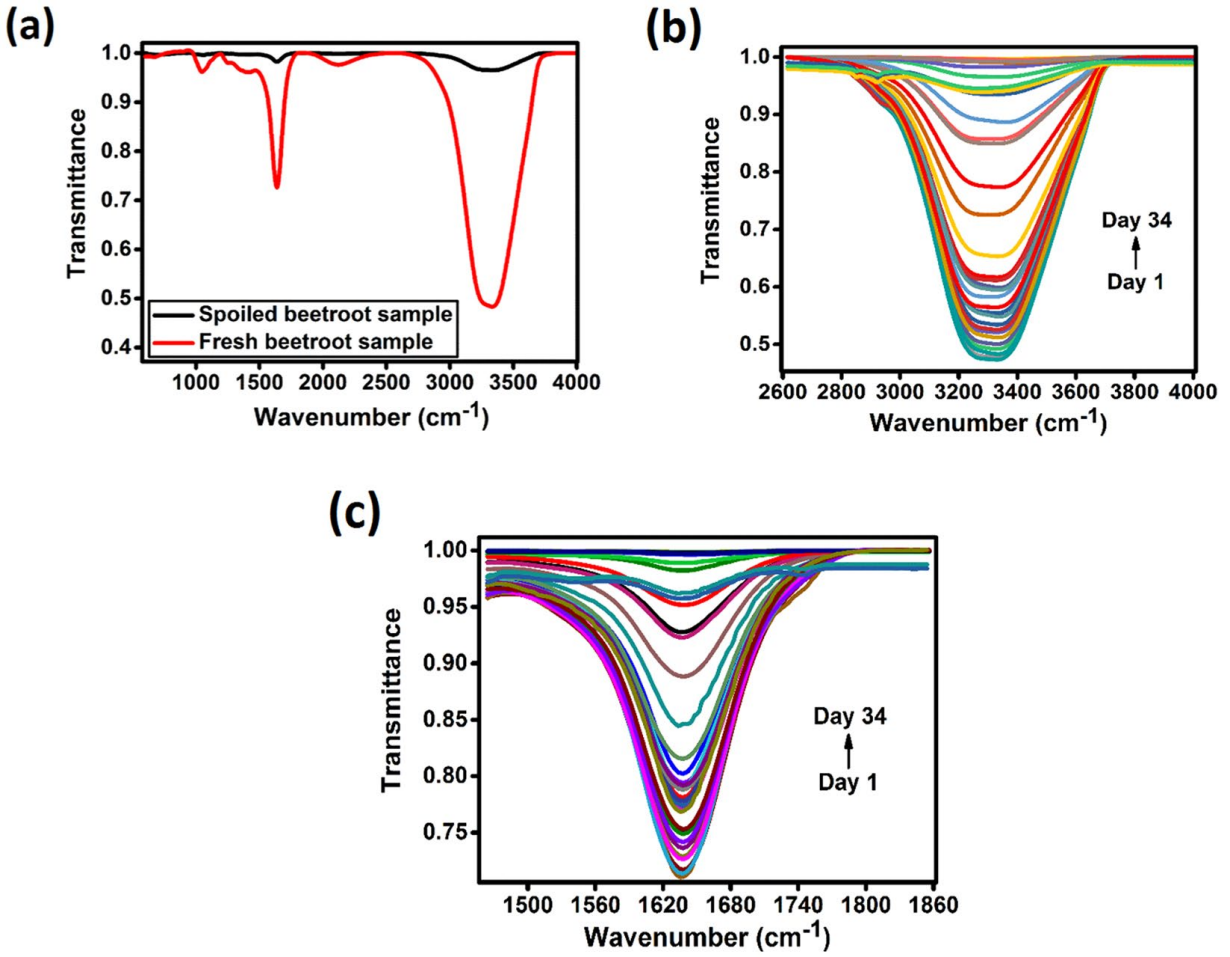
(**a**) FTIR spectra of fresh and spoiled beetroot samples recorded in the spectral range of 400–4000 cm^−1^ and FTIR spectra of beetroot samples recorded for a period of 34 days in the spectral ranges of (**b**) 2614–4000 cm^−1^ and (**c**) 1465–1853 cm^−1^.

The FTIR spectra of fresh and spoiled beetroot test samples recorded in the spectral range of 400–4000 cm^−1^ are shown in Fig. 1(a). As can be seen from Fig. 1(a), the main differences between the FTIR spectra of fresh and spoiled beetroot test samples were observed in the spectral ranges of 2614–4000 cm^−1^ and 1465–1853 cm^−1^. The FTIR spectra of fresh and spoiled beetroot test samples showed wide and strong bands at 3319 and 1637 cm^−1^, which corresponded to asymmetric and symmetric stretching vibrations of OH groups and H-O-H bending arising from the moisture content of beetroot test samples respectively. The results of transmittance at 3319 and 1637 cm^−1^ were in agreement with the results of Kong *et al*.^20^ and Saikia *et al*.^21^. The OH and H-O-H bands of spoiled beetroot test sample became much weaker compared to the OH and H-O-H bands of fresh beetroot test sample. These weak bands were due to the loss of moisture content resulted from the liberation of heat during beetroot respiration. Since the wide and strong bands observed at 3319 and 1637 cm^−1^ were specific to the moisture content of beetroot test samples, FTIR spectra of fresh beetroot test samples were recorded for a period of 34 days in the spectral ranges of 2614–4000 cm^−1^ and 1465–1853 cm^−1^ for the detection of spoilage level of beetroot test samples.

**Figure 2:**
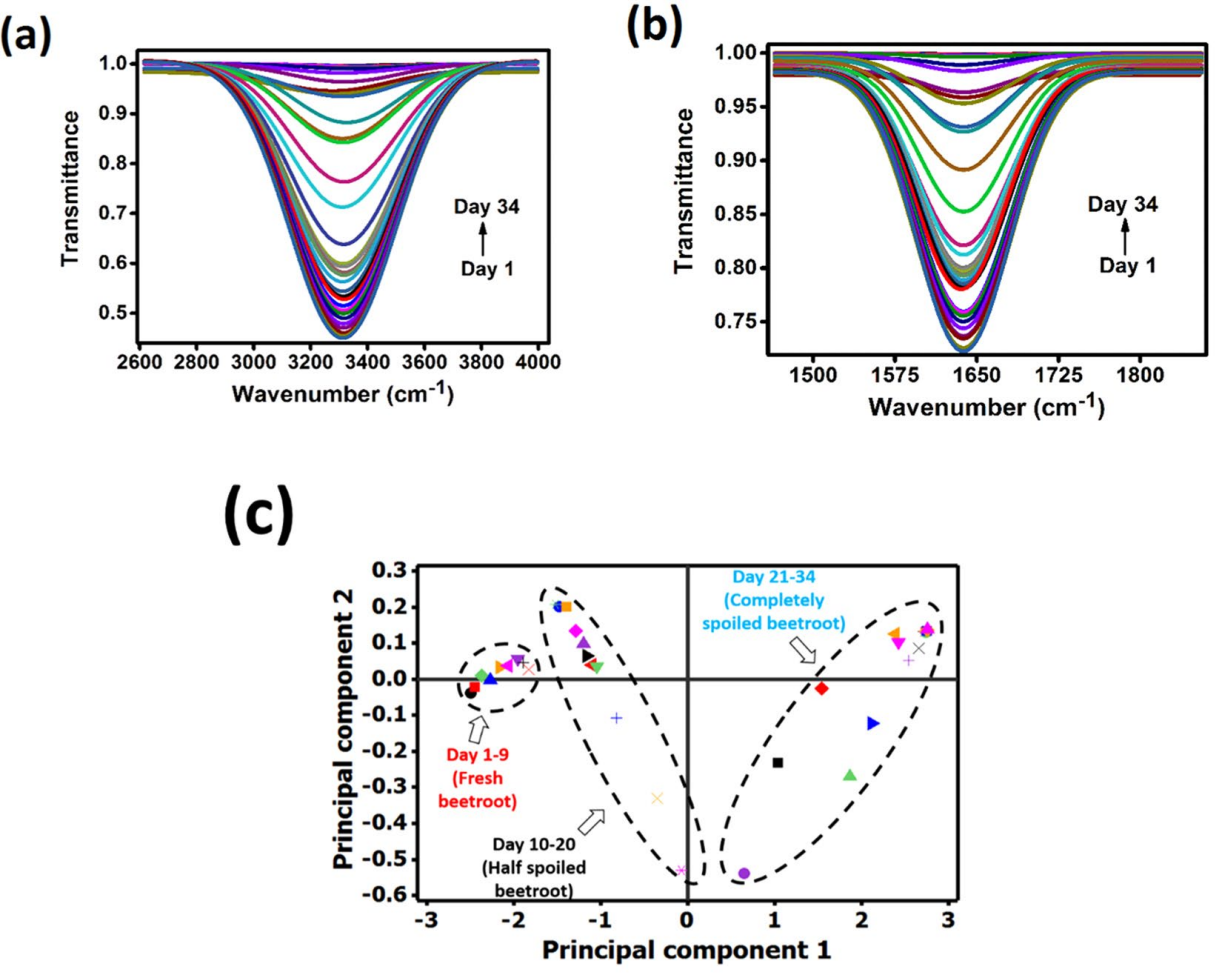
Gaussian fitted FTIR spectra of beetroot samples recorded for a period of 34 days in the spectral ranges of (**a**) 2614–4000 cm^−1^ and (**b**) 1465–1853 cm^−1^ and (**c**) principal component analysis score plot for beetroot quality classification.

Figure 1(b) and (c) show the FTIR spectra of beetroot test samples recorded for a period of 34 days in the spectral ranges of 2614–4000 cm^−1^ and 1465–1853 cm^−1^. The FTIR spectra shown in Fig. 1(b) and (c) displayed differences in transmittance across the mid-infrared regions with the increase in days of beetroot storage. The significant difference in transmittance was due to the loss of moisture content of beetroot test samples. Since the beetroot test samples were stored for a long period of time, it released heat from respiration and subsequently lost moisture, which in-turn decreased the stability and increased susceptibility to decay. Figure 2(a) and (b) show the Gaussian fitted FTIR spectra of beetroot test samples recorded for a period of 34 days in the spectral ranges of 2614–4000 cm^−1^ and 1465–1853 cm^−1^. The height, area, width and centre of the Gaussian peak corresponded to the values of *T*_*p*_, ${\int }_{{\bar{\nu }}_{i}}^{{\bar{\nu }}_{f}}{T}_{p}d\bar{\nu }$, *w* and ${\bar{\nu }}_{c}$ respectively.

The parameters of Gaussian fitted FTIR spectra of beetroot test samples recorded for a period of 34 days in the ranges of 2614–4000 cm^−1^ and 1465–1853 cm^−1^ are given in Supplementary Tables 1 and 2 respectively. The reliability and goodness of fit of Gaussian model were investigated using adjusted regression coefficient. The adjusted R^2^ is calculated as,

1 $$\,{R}^{2}=1-\,\frac{RSS}{TSS},\mathrm{where}\,TSS=\sum _{i=1}^{n}\,{T}_{i}^{2}\,{\rm{and}}\,RSS=\sum _{i=1}^{n}{w}_{i}{({T}_{i}-{T}_{i}^{p})}^{2}$$

where, *w*_*i*_ is the weighted value, *T*_*i*_ and ${T}_{i}^{p}$ are the observed and predicted transmittance, *RSS* is the residual sum of squares and *TSS* is the total sum of squares. The Gaussian model exhibited the high value of adjusted R^2^ in the range of 0.924–0.984. It depicted that the fitting appeared very well and only 1.548–7.511% of total variance was not elucidated by the proposed model. In addition, suitability of the proposed model was examined using reduced chi-square. The reduced chi-square can be determined using equation (2).

2 $$Chi-square=\sum _{i=1}^{n}\frac{{({T}_{i}-{T}_{i}^{p})}^{2}}{{T}_{i}^{p}}$$

The estimated reduced chi-square ranged from 1.01832 × 10^−8^–2.49821 × 10^−4^, displaying the results of the proposed model was satisfactory.

### Discrimination of fresh, half and completely spoiled beetroot samples

FTIR spectroscopy technique coupled to PCA analysis have been employed as a rapid and simple way to determine the moisture level of beetroot test samples. With the aid of PCA, the multidimensional FTIR datasets can be reduced without any significant loss of information. The percentage variance and cumulative variance described by the principal components attained by decomposition of sample FTIR data using PCA are given in Supplementary Table 3. The first column represented the principal component number, the second column displayed the Eigen value, the third column displayed the percentage of variance described by the principal components and the fourth column showed the cumulative variance. As can be seen from Supplementary Table 3 and Fig. 1, 99.99% of the total variance of the sample FTIR data was collected with the two principal components. Based on the analysis, the multidimensional matrix was reduced to two principal components namely principal component 1 (PC1) and principal component 2 (PC2). The score plot for the discrimination of moisture content in fresh, half and completely spoiled beetroot test samples is presented in Fig. 2(c). One can conclude from the score plot that fresh, half and completely spoiled beetroot samples were clearly discriminated from each other with respect to their moisture content. As evident from Fig. 2(c), three well-separated groups were observed. All the fresh beetroot test samples were scattered together in one group at the left side of the score plot, which was represented as day 1–9 in Fig. 2(c). Considering the completely spoiled beetroot test samples, they were scattered together in one group at the right side of the score plot, which was represented as day 21–34 in Fig. 2(c). Similarly, half spoiled beetroot test samples were grouped and favourably located between fresh and completely spoiled beetroot samples at the centre of score plot, which was represented as day 10–20 in Fig. 2(c). In addition, PCA results confirmed that fresh beetroot test samples retained their marketable quality for 9 days at 0 °C. About 50% of the beetroot test samples lost half of its initial moisture content after 20 days whereas, after 34 days of storage, 100% of the beetroot samples lost its initial moisture content completely. Clear separation between the three groups showed individual beetroot test samples with no overlap and allowed the identification of the level of moisture content in beetroot test samples.

### Model calibration and assessment

**Figure 3:**
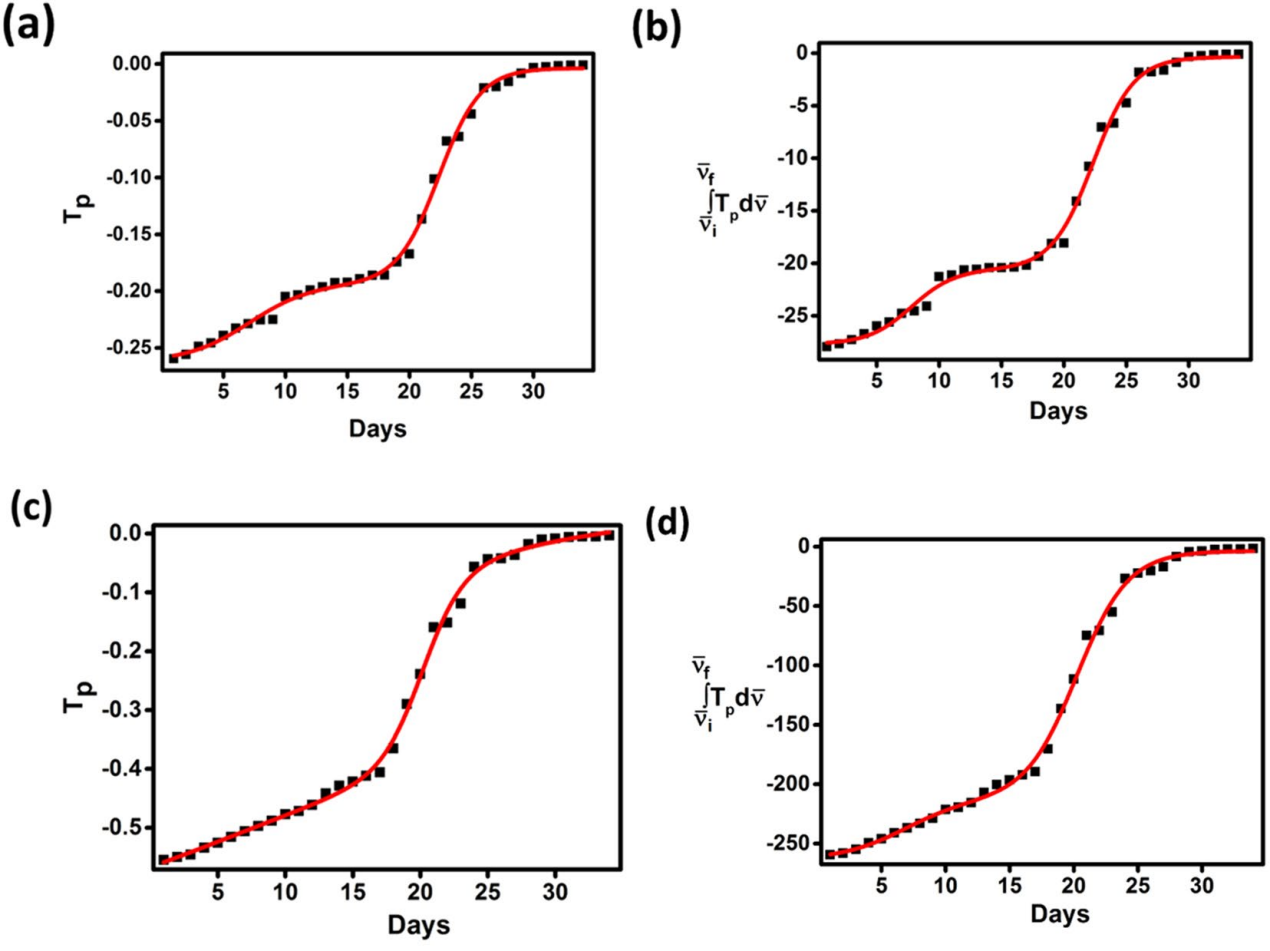
The plots of *T*_*p*_ *vs* days and ${\int }_{{\bar{\nu }}_{i}}^{{\bar{\nu }}_{f}}{T}_{p}d\bar{\nu }$ *vs* days measured in the spectral ranges of 1465–1853 cm^−1^ (**a** and **b**) and 2614–4000 cm^−1^ (**c** and **d**) for the determination of age of beetroot using biphasic dose response model (standard error ≤ 0.01).

Quantitative analysis of age of beetroot test samples was performed with the help of biphasic dose-response model. Figure 3(a–d) show the plots of *T*_*p*_ *vs* days and ${\int }_{{\bar{\nu }}_{i}}^{{\bar{\nu }}_{f}}{T}_{p}d\bar{\nu }$ *vs* days measured in the spectral ranges of 1465–1853 cm^−1^ and 2614–4000 cm^−1^ for the determination of age of beetroot using biphasic dose response model. In the applied spectral range of 1465–1853 cm^−1^ (Fig. 3(a) and (b)), peak transmittance and area under the transmittance curve exhibited noticeable increase in the first 9 days of beetroot storage. Later, both peak transmittance and area under the transmittance curve increased linearly with increasing day of beetroot storage from day 10 to day 19. Finally, peak transmittance and area under the transmittance curve increased in a hyperbolic fashion with the day of beetroot storage from day 20 to day 34. Similarly, in the applied spectral range of 2614–4000 cm^−1^ (Fig. 3(c) and (d)), peak transmittance and area under the transmittance curve increased linearly with increasing day of beetroot storage from day 10 to day 19. At last, peak transmittance and area under the transmittance curve increased in a hyperbolic fashion when the day of beetroot storage increased further (day 20–day 34).

Since the *T*_*p*_ *vs* days and ${\int }_{{\bar{\nu }}_{i}}^{{\bar{\nu }}_{f}}{T}_{p}d\bar{\nu }$ *vs* days curves followed sigmoidal behaviour, biphasic dose response model was introduced in this work. The parameters of the plots of *T*_*p*_ *vs* days and ${\int }_{{\bar{\nu }}_{i}}^{{\bar{\nu }}_{f}}{T}_{p}d\bar{\nu }$ *vs* days for the determination of age of beetroot using biphasic dose response model is given in Table 1. The calibration equations developed using biphasic dose response model were formulated as,

**Table 1:** Parameters of the plots of *T*_*p*_ *vs* days and ${\int }_{{\bar{\nu }}_{i}}^{{\bar{\nu }}_{f}}{T}_{p}d\bar{\nu }$ *vs* days (measured in the range of 1465–1853 cm^−1^ and 2614–4000 cm^−1^) for the determination of age of beetroot using biphasic dose response model.

Parameters	Wavenumber range (cm^−1^)			
	1465–1853		2614–4000	
	***T***_***p***_ *vs* days	${\int }_{{\bar{{\boldsymbol{\nu }}}}_{{\boldsymbol{i}}}}^{{\bar{{\boldsymbol{\nu }}}}_{{\boldsymbol{f}}}}{{\boldsymbol{T}}}_{{\boldsymbol{p}}}d\bar{{\boldsymbol{\nu }}}$ *vs* days	***T***_***p***_ *vs* days	${\int }_{{\bar{{\boldsymbol{\nu }}}}_{{\boldsymbol{i}}}}^{{\bar{{\boldsymbol{\nu }}}}_{{\boldsymbol{f}}}}{{\boldsymbol{T}}}_{{\boldsymbol{p}}}d\bar{{\boldsymbol{\nu }}}$ *vs* days
*T*_*pf*_	−0.262	—	−0.737	—
*T*_*pi*_	−0.003	—	0.050	—
log *day*_1_	7.097	7.796	6.608	6.784
log *day*_2_	22.378	22.325	20.176	20.249
*h*_1_	0.174	0.250	0.033	0.175
*h*_2_	0.259	0.263	0.286	0.216
*p*	0.266	0.261	0.579	0.196
${({\int }_{{\bar{\nu }}_{i}}^{{\bar{\nu }}_{f}}{T}_{p}d\bar{\nu })}_{f}$	—	−27.698	—	−263.643
${({\int }_{{\bar{\nu }}_{i}}^{{\bar{\nu }}_{f}}{T}_{p}d\bar{\nu })}_{i}$	—	−0.362	—	−3.690
R^2^	0.996	0.996	0.997	0.997
RPE (%)	0.033	0.039	0.028	0.028
RMSECV (%)	0.681	0.811	0.580	0.577
Recovery (%)	100.570	100.611	98.625	98.557

*In the applied spectral range of 2614–4000* *cm*^−1^:

3 $${T}_{p}=-\,0.737+0.787[\frac{0.579}{1+{10}^{(6.608-day)0.033}}+\frac{0.421}{1+{10}^{(20.176-day)0.286}}]$$

4 $${\int }_{{\bar{\nu }}_{i}}^{{\bar{\nu }}_{f}}{T}_{p}d\bar{\nu }=-\,263.643+259.953[\frac{0.196}{1+{10}^{(6.784-day)0.175}}+\frac{0.804}{1+{10}^{(20.249-day)0.216}}]$$

*In the applied spectral range of 1465–1853 cm*^*−1*^:

5 $${T}_{p}=-\,0.262+0.259[\frac{0.267}{1+{10}^{(7.097-day)0.174}}+\frac{0.733}{1+{10}^{(22.378-day)0.259}}]$$

6 $${\int }_{{\bar{\nu }}_{i}}^{{\bar{\nu }}_{f}}{T}_{p}d\bar{\nu }=-\,27.698+27.336[\frac{0.261}{1+{10}^{(7.796-day)0.250}}+\frac{0.739}{1+{10}^{(22.325-day)0.263}}]$$

ANOVA was performed to evaluate the suitability and adequacy of the fitted biphasic dose-response model. ANOVA results (Table 2) exhibited that regression coefficients (*T*_*pf*_, *T*_*pi*_, log *day*_1_, log *day*_2_, *h*_1_, *h*_2_, *p*, ${({\int }_{{\bar{\nu }}_{i}}^{{\bar{\nu }}_{f}}{T}_{p}d\bar{\nu })}_{f}$ and ${({\int }_{{\bar{\nu }}_{i}}^{{\bar{\nu }}_{f}}{T}_{p}d\bar{\nu })}_{i}$) were significant (p < 0.05 with 95% confidence interval). It also displayed that the regression coefficients had significant effect on the peak transmittance and area under the transmittance curve. In addition, the calculated F-values were significantly high, suggesting that the proposed biphasic dose response model was satisfactory. One can conclude from the ANOVA result that the parameters namely *T*_*pf*_, *T*_*pi*_, $\mathrm{log}\,da{y}_{1}$, log *day*_1_, log *day*_2_, *h*_1_, *h*_2_, *p*, ${({\int }_{{\bar{\nu }}_{i}}^{{\bar{\nu }}_{f}}{T}_{p}d\bar{\nu })}_{f}$ and ${({\int }_{{\bar{\nu }}_{i}}^{{\bar{\nu }}_{f}}{T}_{p}d\bar{\nu })}_{i}$ were dependent on the peak transmittance and area under the transmittance curve.

**Table 2:** Analysis of variance results of *T*_*p*_ *vs* days and ${\int }_{{\bar{\nu }}_{i}}^{{\bar{\nu }}_{f}}{T}_{p}d\bar{\nu }$ *vs* days models measured in the ranges of 1465–1853 cm^−1^ and 2614–4000 cm^−1^ for the determination of age of beetroot using biphasic dose response model.

Model	Degrees of freedom	Sum of squares	Mean square	F value	Probability >F
*T*_*p*_ *vs* days (1465–1853cm^−1^)	7	0.958	0.137	4633.049	0
Residual	27	7.983 × 10^−4^	2.956 × 10^−5^	—	—
Uncorrected Total	34	0.959	—	—	—
Corrected Total	33	0.299	—	—	—
${\int }_{{\bar{\nu }}_{i}}^{{\bar{\nu }}_{f}}{T}_{p}d\bar{\nu }$ *vs* days (1465–1853cm^−1^)	7	10991.671	1570.238	3736.052	0
Residual	27	11.347	0.420	—	—
Uncorrected Total	34	11003.019	—	—	—
Corrected Total	33	3489.368	—	—	—
*T*_*p*_ *vs* days (2614–4000 cm^−1^)	7	4.374	0.624	5425.878	0
Residual	27	0.003	1.151 × 10^−4^	—	—
Uncorrected Total	34	4.377	—	—	—
Corrected Total	33	1.554	—	—	—
${\int }_{{\bar{\nu }}_{i}}^{{\bar{\nu }}_{f}}{T}_{p}d\bar{\nu }$ *vs* days (2614–4000 cm^−1^)	7	955315.187	136473.598	5119.528	0
Residual	27	719.751	26.657	—	—
Uncorrected Total	34	956034.938	—	—	—
Corrected Total	33	339269.079	—	—	—

In order to assess the goodness of the fitted biphasic dose-response model, the residual analysis was performed. Supplementary Fig. 2(a–d) show the regular residual plots of *T*_*p*_ *vs* days and ${\int }_{{\bar{\nu }}_{i}}^{{\bar{\nu }}_{f}}{T}_{p}d\bar{\nu }$ *vs* days measured in the spectral ranges of 1465–1853 cm^−1^ (Supplementary Fig. 2(a) and (b)) and 2614–4000 cm^−1^ (Supplementary Fig. 2(c) and (d)) for the determination of age of beetroot using biphasic dose response model. As can be seen from Supplementary Fig. 2(a–d), the data points were randomly dispersed around the horizontal axis (f(days) = 0), assuring that fitted biphasic dose-response model was acceptable and reliable for the determination of age of beetroot samples.

To evaluate the accuracy of the fitted biphasic dose-response model, RPE, RMSECV and % recovery were calculated. The calibrated model showing good prediction accuracy has low RPE, RMSECV and 100% recovery. The error analysis results exhibited that fitted biphasic dose-response function over the spectral range of 2614–4000 cm^−1^ displayed small RPE (RPE(*T*_*p*_ *vs* days) = 0.028 & RPE(${\int }_{{\bar{\nu }}_{i}}^{{\bar{\nu }}_{f}}{T}_{p}d\bar{\nu }$ *vs* days) = 0.028) and RMSECV (RMSECV(*T*_*p*_ *vs* days) = 0.580 & RMSECV(${\int }_{{\bar{\nu }}_{i}}^{{\bar{\nu }}_{f}}{T}_{p}d\bar{\nu }$ *vs* days) = 0.577) whereas, fitted biphasic dose-response function over the spectral range of 1465–1853 cm^−1^ displayed large RPE (RPE(*T*_*p*_ *vs* days) = 0.033 & RPE(${\int }_{{\bar{\nu }}_{i}}^{{\bar{\nu }}_{f}}{T}_{p}d\bar{\nu }$ *vs* days) = 0.039) and RMSECV (RMSECV(*T*_*p*_ *vs* days) = 0.681 & RMSECV(${\int }_{{\bar{\nu }}_{i}}^{{\bar{\nu }}_{f}}{T}_{p}d\bar{\nu }$ *vs* days) = 0.811). All these validation results suggested that the proposed biphasic dose-response model over the spectral range of 2614–4000 cm^−1^ can predict the age of beetroot samples with good accuracy.

### Analytical application

**Figure 4:**
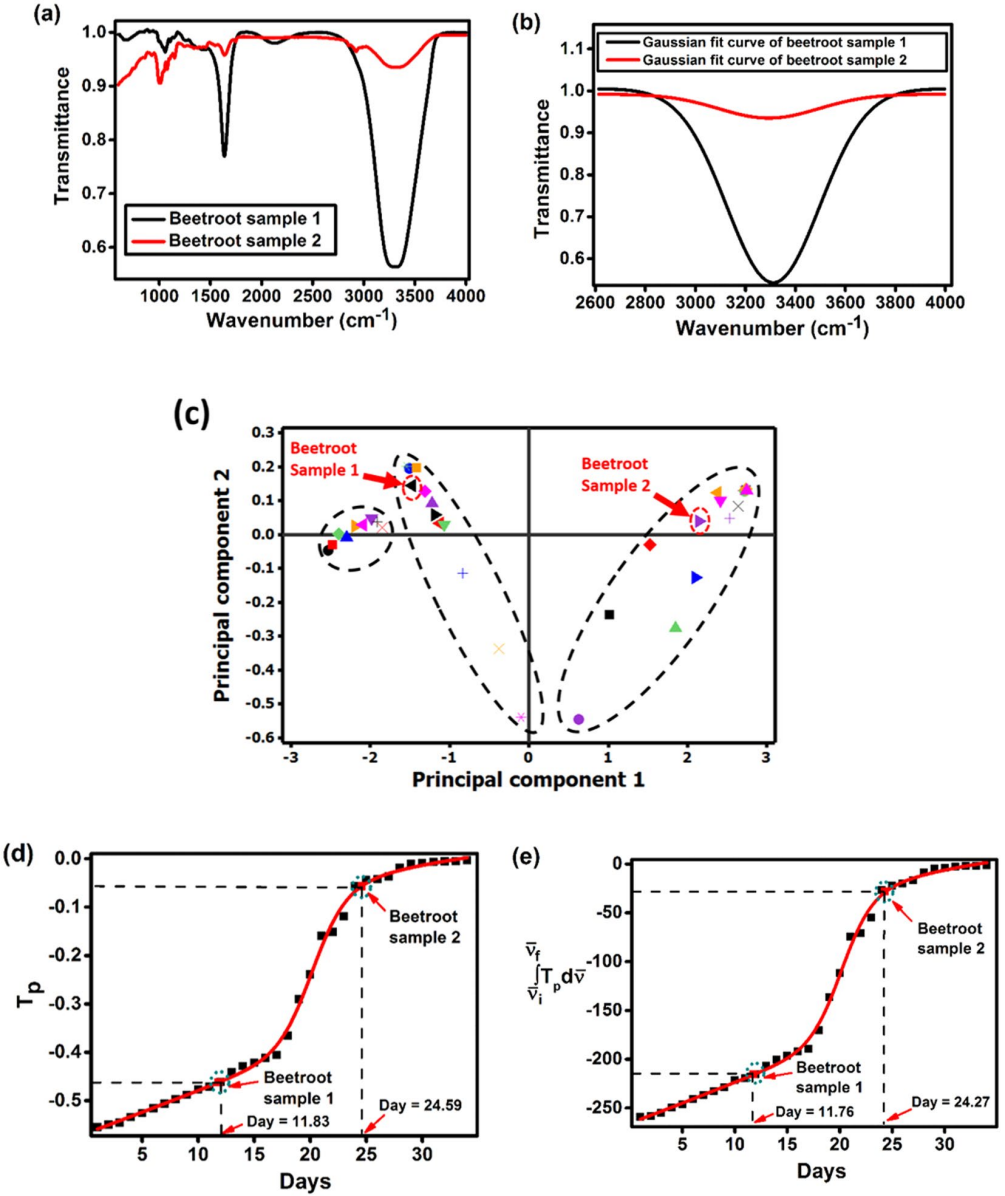
(**a**) FTIR spectra of beetroot samples 1 and 2 measured in the spectral range of 600–4000 cm^−1^, (**b**) Gauss fitted FTIR spectra of beetroot samples 1 and 2 recorded in the spectral range of 2614–4000 cm^−1^, (**c**) principal component analysis score plot for beetroot quality classification and determination of age of beetroot using biphasic dose response model: (**d**) *T*_p_ *vs* days and (**e**) ${\int }_{{\bar{\nu }}_{i}}^{{\bar{\nu }}_{f}}{T}_{p}d\bar{\nu }$ *vs* days.

In order to study the practicability of the proposed analytical method, two freshly harvested beetroots were collected and refrigerated at 0 °C. For the measurements, beetroot samples after 12 and 25 days of storage were taken, which were represented as beetroot sample 1 and 2 respectively in Fig. 4(a). Later, the beetroot samples were cut into small pieces (3 × 4 × 2 cm) and ATR-FTIR spectra were collected for each beetroot sample (Fig. 4(a)). After normalizing the FTIR spectra, Gaussian curve fitting algorithm was applied on FTIR datasets (Fig. 4(b)). The formulated Gaussian fitted FTIR spectra of beetroot samples after 12 and 25 days of storage were obtained as,

*For beetroot sample 1*:

7 $$T=1.005+\frac{-215.511}{372.023\sqrt{\pi /2}}{e}^{-2\frac{{(\bar{\nu }-3311.304)}^{2}}{{372.023}^{2}}}$$

*For beetroot sample 2*:

8 $$T=0.992+\frac{-28.356}{398.339\sqrt{\pi /2}}{e}^{-2\frac{{(\bar{\nu }-3293.512)}^{2}}{{398.339}^{2}}}$$

Figure 4(c) shows the score plot for beetroot quality classification. The beetroot sample 1 was circled in red colour and marked with black colour on the score plot and it was located at the left side of the score plot. Regarding the beetroot sample 2, it was circled in red colour and marked with violet colour on the score plot. The beetroot sample 2 was located at the right side of the score plot. All these score plot results indicated that the beetroot sample 1 lost half of its moisture content whereas beetroot sample 2 lost its moisture content completely.

In order to predict the age of beetroot sample 1 and 2, the calibrated biphasic dose-response models (Eqs 3 and 4) were used. The fitted biphasic dose-response models over the datasets of T_p_ = f(days) (Fig. 4(d)) and ${\int }_{{\bar{\nu }}_{i}}^{{\bar{\nu }}_{f}}{T}_{p}d\bar{\nu }$ = f(days) (Fig. 4(e)) determined the ages of beetroot sample 1 and 2 as 11.83, 11.76 days and 24.59, 24.27 days respectively with an accuracy of 97.5%. All these results suggest that PCA combined with FTIR technique can determine the age and moisture level of beetroot with enhanced accuracy.

## Conclusion

In this study, the age of beetroot and discrimination of moisture content in fresh, half and completely spoiled beetroot samples were successfully performed by coupling ATR-FTIR technique with PCA analysis. The developed biphasic dose-response model accurately determined the age of beetroot samples with a very minimal RPE and RMSECV. The proposed analytical method doesn’t require sample pre-treatment. One of the advantages of this work is that the ATR-FTIR technique coupled with PCA works accurately without any false positive results when the beetroot is refrigerated at 0 °C. Moreover, the proposed analytical method is rapid, simple, easy to operate and suitable for continuous monitoring of moisture content during production, storage, distribution and transportation of beetroot.

## Materials and Methods

### Sample preparation

A total of ten beetroots (*Beta vulgaris L*.) samples were collected from a local supermarket in Thanjavur, India. In this research, two beetroot samples were employed to test and validate the proposed beetroot quality predictive models in investigating the moisture content and ages of beetroot samples. For the measurements, beetroot samples were cut into small pieces (3 × 4 × 2 cm) and spectra were collected for each beetroot sample.

### FTIR measurement

The FTIR spectra of beetroot samples were recorded on Alpha T FTIR spectrometer (Bruker, Germany) using attenuated total reflectance (ATR) in the spectral ranges of 2614–4000 cm^−1^ and 1465–1853 cm^−1^ with a spectral resolution of 8 cm^−1^. Frequent FTIR measurements were recorded directly from the beetroot sample surface over a period of 34 days. During each measurement, the FTIR spectra were normalized. Prior to every single measurement, a background air spectrum was scanned in order to minimize baseline correction. The background spectrum was then subtracted from the sample spectrum. After each measurement, the sample holder in the FTIR was cleaned with ethanol and distilled water. All data processing and manipulation of FTIR spectra were carried out using MATLAB 2016b software.

### Establishing calibration equation

In order to estimate transmittance peak height and area under the transmittance curve over the spectral ranges of 2614–4000 cm^−1^ and 1465–1853 cm^−1^, Gaussian curve fitting algorithm was performed on the collected FTIR data. The Gaussian function is capable of describing the dependencies between transmittance and wavenumber. The parameters namely transmittance peak height and area under the transmittance curve were calculated using the relation (equation (9)),

9 $$T={T}_{0}+\frac{{\int }_{{\bar{\nu }}_{i}}^{{\bar{\nu }}_{f}}{T}_{p}d\bar{\nu }}{w\sqrt{\pi /2}}{e}^{-2\frac{{(\bar{\nu }-{\bar{\nu }}_{c})}^{2}}{{w}^{2}}}$$

where, *w* is the full width at half maximum of the transmittance peak height, *T* is the transmittance, *T*_*p*_ is the transmittance peak height, *T*_0_ is the baseline transmittance, ${\bar{\nu }}_{c}$ is the wavenumber at which maximum transmittance was observed and ${\bar{\nu }}_{i}$ & ${\bar{\nu }}_{f}$ are the initial and final wavenumber. The coefficients *T*_*p*_, ${\int }_{{\bar{\nu }}_{i}}^{{\bar{\nu }}_{f}}{T}_{p}d\bar{\nu }$ and *w* of the proposed Gaussian function were estimated by means of Levenberg-Marquardt algorithm.

The shapes of *T*_*p*_ *vs* days and ${\int }_{{\bar{\nu }}_{i}}^{{\bar{\nu }}_{f}}{T}_{p}d\bar{\nu }$ *vs* days curves were similar to biphasic dose response curve. Hence, non-linear biphasic dose response models (equations (10) and (11)) were fitted to the experimental results.

10 $${T}_{p}={T}_{pf}+({T}_{pi}-{T}_{pf})[\frac{p}{1+{10}^{(\mathrm{log}da{y}_{1}-day){h}_{1}}}+\frac{1-p}{1+{10}^{(\mathrm{log}da{y}_{2}-day){h}_{2}}}]$$

11 $${\int }_{{\bar{\nu }}_{i}}^{{\bar{\nu }}_{f}}{T}_{p}d\bar{\nu }={({\int }_{{\bar{\nu }}_{i}}^{{\bar{\nu }}_{f}}{T}_{p}d\bar{\nu })}_{f}+[{({\int }_{{\bar{\nu }}_{i}}^{{\bar{\nu }}_{f}}{T}_{p}d\bar{\nu })}_{i}-{({\int }_{{\bar{\nu }}_{i}}^{{\bar{\nu }}_{f}}{T}_{p}d\bar{\nu })}_{f}]\,[\frac{p}{1+{10}^{(\mathrm{log}da{y}_{1}-day){h}_{1}}}+\frac{1-p}{1+{10}^{(\mathrm{log}da{y}_{2}-day){h}_{2}}}]$$

where, *T*_*pf*_ and *T*_*pi*_ are the peak transmittance at day 34 and day 1 respectively, ${({\int }_{{\bar{\nu }}_{i}}^{{\bar{\nu }}_{f}}{T}_{p}d\bar{\nu })}_{f}\,$and ${({\int }_{{\bar{\nu }}_{i}}^{{\bar{\nu }}_{f}}{T}_{p}d\bar{\nu })}_{i}\,$are the area under the transmittance curve at day 34 and day 1 respectively, *h*_1_ and *h*_2_ are the slopes of the curves, *p* is the proportion and $\mathrm{log}\,da{y}_{1}$ and $\mathrm{log}\,da{y}_{2}$ are the days where the response (*T*_*p*_ or ${\int }_{{\bar{\nu }}_{i}}^{{\bar{\nu }}_{f}}{T}_{p}d\bar{\nu }$) is reduced by half. In addition, analysis of variance (ANOVA) was carried out to evaluate the existence of statistical difference between the regression coefficients. The probability value less than 0.05 was considered significant.

### Qualitative principal component analysis

In this work, PCA, an unsupervised method of multivariate analysis, was employed to represent the variations presents in beetroot samples using the smallest number of principal components. PCA of mid-infrared FTIR spectral data was performed in order to sort beetroot samples according to their moisture levels as well as to monitor the interrelated clusters and sub-clusters in which the beetroot samples can be scattered. Beetroot samples were sorted into three groups based on its moisture content. In the score plot of PCA, two principal components were taken into account to allow the graphical display of whole FTIR dataset.

### Validation of results

The predictive ability of proposed biphasic dose-response models to correctly identify the age of the beetroot from the day on which it has been refrigerated immediately after harvesting was compared and validated by estimating relative prediction error (RPE) (equation (12)), percentage recovery (% recovery) (equation (13)) and root mean square error of cross-validation (RMSECV) (equation (14)).

12 $$RP{E}_{S}={[\frac{{\sum }_{i=1}^{n}{(Dayofstorag{e}_{(predicted)}-Dayofstorag{e}_{(observed)})}^{2}}{{\sum }_{i=1}^{n}{(Dayofstorag{e}_{(predicted)})}^{2}}]}^{0.5}$$

13 $$ \% \,recovery=100\times \,[\frac{1}{n}\sum _{i=1}^{n}\frac{Day\,of\,storag{e}_{(predicted)}}{Day\,of\,storag{e}_{(observed)}}]$$

14 $${RMSECV}={[\frac{1}{n}\sum _{i=1}^{n}{(Dayofstorag{e}_{(predicted)}-Dayofstorag{e}_{(observed)})}^{2}]}^{0.5}$$

where, $Day\,of\,storag{e}_{(predicted)}$ and $Day\,of\,storag{e}_{(observed)}$ are the predicted and observed ages of beetroot from the day on which it has been refrigerated immediately after harvesting and *n* is the number of samples. And also, adjusted regression coefficients (R^2^) between the predicted and observed values of *T*_*p*_ and ${\int }_{{\bar{\nu }}_{i}}^{{\bar{\nu }}_{f}}{T}_{p}d\bar{\nu }$ were calculated to validate beetroot quality predictive models (*T*_*p*_ = f(days) and ${\int }_{{\bar{\nu }}_{i}}^{{\bar{\nu }}_{f}}{T}_{p}d\bar{\nu }$ = f(days)) for the effective determination of spoilage level of beetroot. Quantitative analysis of sample FTIR data, Gaussian curve fitting, ANOVA, PCA and nonlinear regression analyses were performed with MATLAB 2016b.

## Electronic supplementary material

Supplementary Information
