# Supplementary material for: Analysis of Moisture Content in Beetroot using Fourier Transform Infrared Spectroscopy and by Principal Component Analysis
Source: Sci Rep. 2018 May 22;8:7996. doi: 10.1038/s41598-018-26243-5 (PMC5964165; doi:10.1038/s41598-018-26243-5)
Supplement: Supplementary file 1 — Supplementary Information [file 41598_2018_26243_MOESM1_ESM.docx]

**Analysis of Moisture Content in Beetroot using Fourier Transform Infrared Spectroscopy and by Principal Component Analysis**

Noel Nesakumar^a,**^, Chanthini Baskar^b,**^, Srinivasan Kesavan^a^, John Bosco Balaguru Rayappan^c,#^ and Subbiah Alwarappan^a,#^

*^a^Electrodics and Electrocatalysis Division, CSIR-Central Electrochemical Research Institute, Karaikudi 630003, Tamil Nadu, India*

*^b^School of Computing, SASTRA Deemed University and ^c^School of Electrical and Electronics Engineering, SASTRA Deemed University, Thanjavur 613401, Tamil Nadu, India*

*****Both authors contributed equally to this work***

^#^ Corresponding Author (s): Dr Subbiah Alwarappan (e.mail: [alwarappan@cecri.res.in](mailto:alwarappan@cecri.res.in))

Dr John Bosco Balaguru Rayappan (e.mail: [rjbosco@ece.sastra.edu](mailto:rjbosco@ece.sastra.edu))


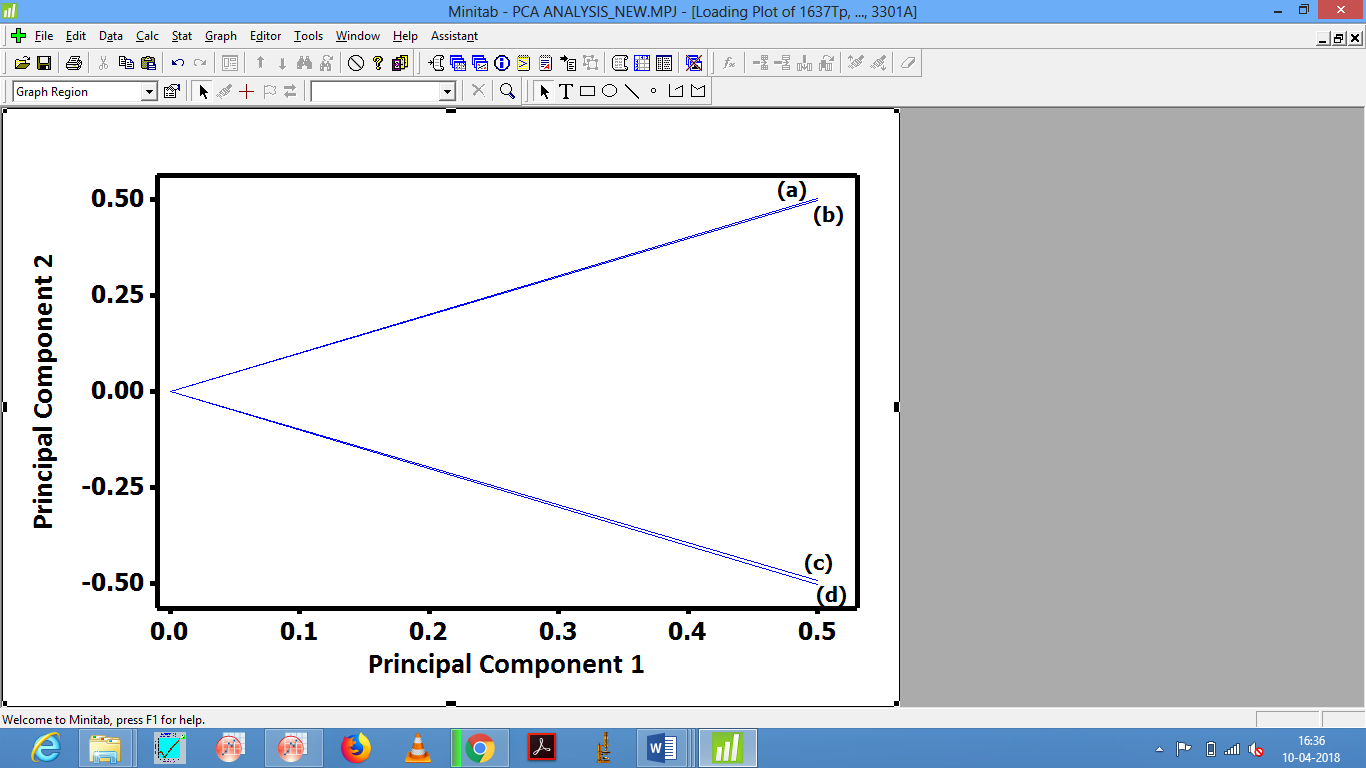
**Supplementary Figure 1.** PCA loading plot for the collected sample FTIR data. (a) represents $T_{p}$ measured in the spectral range of 1465 – 1853 cm^-1^, (b) represents $\int_{\overline{\nu}_{i}}^{\overline{\nu}_{f}} T_{p}d\overline{\nu}$ measured in the spectral range of 1465 – 1853 cm^-1^, (c) represents $\int_{\overline{\nu}_{i}}^{\overline{\nu}_{f}} T_{p}d\overline{\nu}$ measured in the spectral range of 2614 – 4000 cm^-1^ and (d) represents $T_{p}$ measured in the spectral range of 2614 – 4000 cm^-1^.

**Supplementary Figure 2.** The regular residual plots of $T_{p}$ *vs* days and $\int_{\overline{\nu}_{i}}^{\overline{\nu}_{f}} T_{p}d\overline{\nu}$ *vs* days measured in the spectral range of 1465 – 1853 cm^-1^ (a & b) and 2614 – 4000 cm^-1^ (c & d) for the determination of age of beetroot using biphasic dose response model (standard error ≤ 0.01).

**
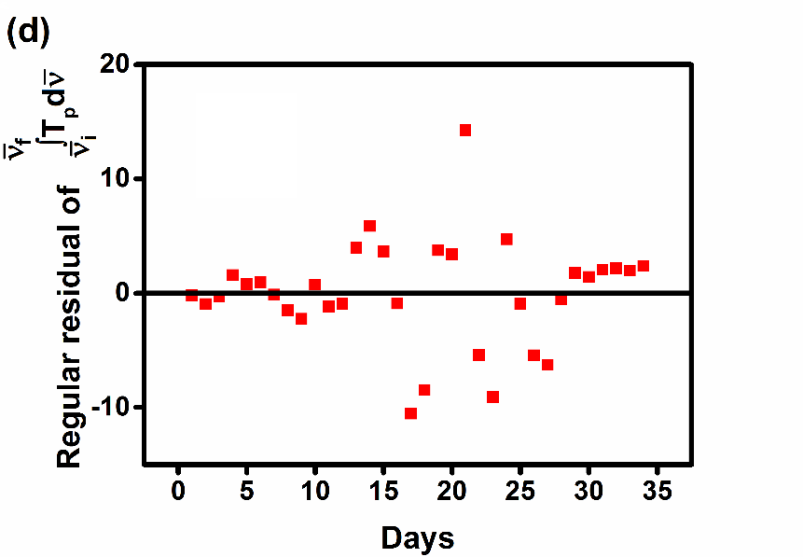

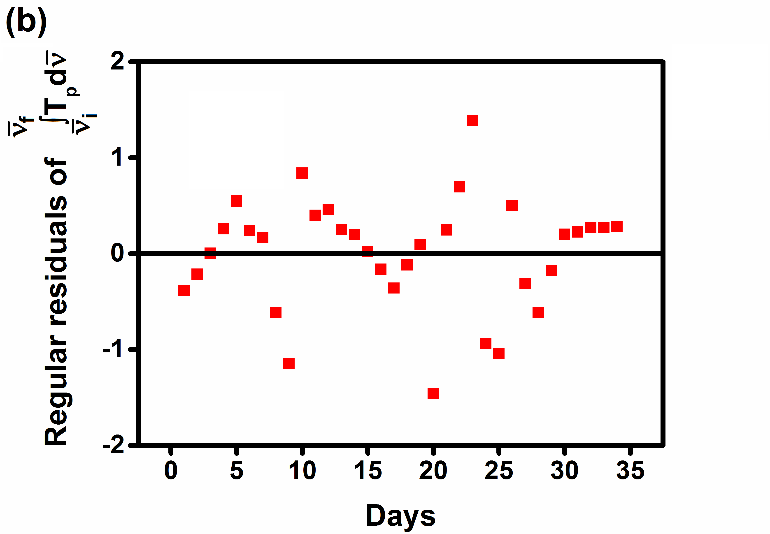




**

**Supplementary Table 1.** Parameters of Gaussian fitted FTIR spectra of beetroot samples recorded for a period of 34 days in the range of 2614 – 4000 cm^-1^.

| **Days** | $\boldsymbol{T}_{\boldsymbol{0}}$ | $\boldsymbol{w}$ | $\int_{\overline{\nu}_{i}}^{\overline{\nu}_{f}} T_{p}d\overline{\nu}$ | ${\overline{\boldsymbol{\nu}}}_{\boldsymbol{c}}$ | $\boldsymbol{T}_{\boldsymbol{p}}$ | **R^2^** | **Reduced chi-square** |
| --- | --- | --- | --- | --- | --- | --- | --- |
|  | **(a.u.)** | **(cm^-1^)** | **(cm^-1^)** | **(cm^-1^)** | **(a.u.)** |  |  |
| Day 1 | 1.004 | 373.028 | -259.320 | 3312.581 | -0.554 | 0.963 | 2.342×10^-4^ |
| Day 2 | 1.004 | 374.454 | -258.127 | 3313.838 | -0.550 | 0.963 | 2.352×10^-4^ |
| Day 3 | 1.006 | 372.627 | -254.822 | 3317.013 | -0.545 | 0.964 | 2.013×10^-4^ |
| Day 4 | 1.004 | 372.684 | -249.454 | 3314.852 | -0.534 | 0.964 | 2.121×10^-4^ |
| Day 5 | 1.004 | 373.371 | -246.019 | 3315.022 | -0.525 | 0.963 | 2.101×10^-4^ |
| Day 6 | 1.005 | 372.781 | -240.963 | 3316.607 | -0.515 | 0.964 | 1.877×10^-4^ |
| Day 7 | 1.005 | 373.230 | -236.798 | 3315.198 | -0.506 | 0.964 | 1.806×10^-4^ |
| Day 8 | 1.003 | 373.765 | -232.959 | 3314.555 | -0.497 | 0.963 | 2.113×10^-4^ |
| Day 9 | 1.003 | 373.998 | -228.819 | 3314.748 | -0.488 | 0.963 | 1.961×10^-4^ |
| Day 10 | 1.005 | 370.309 | -221.440 | 3317.501 | -0.477 | 0.964 | 1.528×10^-4^ |
| Day 11 | 1.005 | 371.380 | -219.503 | 3314.277 | -0.471 | 0.964 | 1.502×10^-4^ |
| Day 12 | 1.005 | 372.889 | -215.599 | 3312.111 | -0.461 | 0.964 | 1.440×10^-4^ |
| Day 13 | 1.004 | 373.521 | -206.869 | 3311.101 | -0.441 | 0.964 | 1.339×10^-4^ |
| Day 14 | 1.004 | 372.983 | -200.395 | 3317.067 | -0.428 | 0.964 | 1.269×10^-4^ |
| Day 15 | 1.003 | 371.641 | -196.474 | 3318.338 | -0.421 | 0.964 | 1.318×10^-4^ |
| Day 16 | 1.00 | 372.375 | -192.241 | 3313.985 | -0.411 | 0.964 | 1.131×10^-4^ |
| Day 17 | 1.004 | 372.363 | -189.413 | 3314.170 | -0.405 | 0.964 | 1.133×10^-4^ |
| Day 18 | 1.003 | 371.773 | -170.271 | 3315.797 | -0.365 | 0.964 | 9.510×10^-5^ |
| Day 19 | 1.002 | 374.874 | -136.239 | 3310.644 | -0.289 | 0.964 | 6.178×10^-5^ |
| Day 20 | 1.002 | 372.490 | -111.433 | 3316.770 | -0.238 | 0.963 | 4.479×10^-5^ |
| Day 21 | 1.001 | 373.640 | -74.649 | 3312.760 | -0.159 | 0.964 | 1.811×10^-5^ |
| Day 22 | 1.001 | 372.723 | -70.672 | 3311.623 | -0.151 | 0.964 | 1.580×10^-5^ |
| Day 23 | 1.001 | 368.767 | -55.031 | 3326.692 | -0.119 | 0.963 | 1.125×10^-5^ |
| Day 24 | 0.990 | 377.718 | -26.803 | 377.718 | -0.056 | 0.966 | 3.161×10^-5^ |
| Day 25 | 0.983 | 409.595 | -22.444 | 3281.527 | -0.043 | 0.964 | 3.423×10^-5^ |
| Day 26 | 0.987 | 384.624 | -20.341 | 3276.145 | -0.042 | 0.961 | 1.648×10^-5^ |
| Day 27 | 1 | 371.015 | -16.900 | 3317.197 | -0.036 | 0.963 | 1.113×10^-6^ |
| Day 28 | 0.999 | 372.717 | -8.495 | 3311.296 | -0.018 | 0.962 | 3.228×10^-7^ |
| Day 29 | 1 | 356.160 | -4.546 | 3359.281 | -0.010 | 0.967 | 1.624×10^-7^ |
| Day 30 | 0.999 | 360.535 | -3.881 | 3343.087 | -0.008 | 0.968 | 1.103×10^-7^ |
| Day 31 | 0.999 | 331.036 | -2.623 | 3374.834 | -0.006 | 0.965 | 7.176×10^-8^ |
| Day 32 | 0.999 | 322.465 | -2.117 | 3386.344 | -0.005 | 0.967 | 7.776×10^-8^ |
| Day 33 | 0.999 | 336.099 | -2.099 | 3375.669 | -0.004 | 0.960 | 2.925×10^-8^ |
| Day 34 | 0.999 | 336.099 | -2.099 | 3375.669 | -0.004 | 0.960 | 2.925×10^-8^ |

**Supplementary Table 2.** Parameters of Gaussian fitted FTIR spectra of beetroot samples recorded for a period of 34 days in the range of 1465 – 1853 cm^-1^.

| **Days** | $\boldsymbol{T}_{\boldsymbol{0}}$ | $\boldsymbol{w}$ | $\int_{\overline{\nu}_{i}}^{\overline{\nu}_{f}} T_{p}d\overline{\nu}$ | ${\overline{\boldsymbol{\nu}}}_{\boldsymbol{c}}$ | $\boldsymbol{T}_{\boldsymbol{p}}$ | **R^2^** | **Reduced chi-square** |
| --- | --- | --- | --- | --- | --- | --- | --- |
|  | **(a.u.)** | **(cm^-1^)** | **(cm^-1^)** | **(cm^-1^)** | **(a.u.)** |  |  |
| Day 1 | 0.982 | 85.040 | -27.947 | 1637.785 | -0.259 | 0.971 | 2.363×10^-4^ |
| Day 2 | 0.981 | 87.131 | -27.670 | 1637.709 | -0.255 | 0.969 | 2.498×10^-4^ |
| Day 3 | 0.983 | 87.533 | -27.272 | 1637.679 | -0.248 | 0.968 | 2.411×10^-4^ |
| Day 4 | 0.982 | 86.781 | -26.721 | 1637.793 | -0.245 | 0.968 | 2.384×10^-4^ |
| Day 5 | 0.983 | 86.656 | -25.965 | 1637.706 | -0.239 | 0.966 | 2.398×10^-4^ |
| Day 6 | 0.983 | 87.726 | -25.587 | 1637.723 | -0.232 | 0.967 | 2.159×10^-4^ |
| Day 7 | 0.984 | 85.587 | -24.765 | 1637.743 | -0.228 | 0.968 | 2.026×10^-4^ |
| Day 8 | 0.984 | 87.677 | -24.533 | 1637.857 | -0.225 | 0.967 | 2.023×10^-4^ |
| Day 9 | 0.984 | 85.404 | -24.084 | 1637.826 | -0.225 | 0.968 | 1.948×10^-4^ |
| Day 10 | 0.985 | 82.917 | -21.276 | 1635.670 | -0.204 | 0.966 | 1.719×10^-4^ |
| Day 11 | 0.984 | 82.880 | -21.115 | 1637.658 | -0.203 | 0.964 | 1.794×10^-4^ |
| Day 12 | 0.984 | 82.421 | -20.644 | 1637.740 | -0.199 | 0.961 | 1.865×10^-4^ |
| Day 13 | 0.984 | 82.086 | -20.582 | 1637.743 | -0.196 | 0.965 | 1.613×10^-4^ |
| Day 14 | 0.985 | 84.524 | -20.430 | 1637.743 | -0.192 | 0.964 | 1.635×10^-4^ |
| Day 15 | 0.986 | 85.541 | -20.418 | 1637.780 | -0.192 | 0.967 | 1.494×10^-4^ |
| Day 16 | 0.986 | 82.989 | -20.363 | 1637.816 | -0.189 | 0.963 | 1.554×10^-4^ |
| Day 17 | 0.986 | 86.087 | -20.191 | 1637.734 | -0.186 | 0.965 | 1.541×10^-4^ |
| Day 18 | 0.986 | 87.513 | -19.337 | 1637.740 | -0.185 | 0.966 | 1.444×10^-4^ |
| Day 19 | 0.986 | 82.853 | -18.111 | 1637.810 | -0.174 | 0.962 | 1.386×10^-4^ |
| Day 20 | 0.988 | 86.147 | -18.073 | 1637.736 | -0.167 | 0.967 | 1.135×10^-4^ |
| Day 21 | 0.988 | 82.300 | -14.077 | 1637.897 | -0.136 | 0.959 | 9.214×10^-5^ |
| Day 22 | 0.992 | 84.798 | -10.763 | 1637.787 | -0.101 | 0.963 | 4.619×10^-5^ |
| Day 23 | 0.994 | 82.788 | -7.039 | 1637.675 | -0.067 | 0.958 | 2.326×10^-5^ |
| Day 24 | 0.995 | 83.172 | -6.666 | 1637.755 | -0.063 | 0.962 | 1.894×10^-5^ |
| Day 25 | 0.997 | 85.859 | -4.730 | 1637.817 | -0.043 | 0.967 | 7.805×10^-6^ |
| Day 26 | 0.979 | 69.305 | -1.821 | 1638.694 | -0.020 | 0.963 | 1.465×10^-5^ |
| Day 27 | 0.983 | 71.821 | -1.794 | 1638.019 | -0.019 | 0.953 | 1.423×10^-5^ |
| Day 28 | 0.998 | 82.332 | -1.601 | 1637.773 | -0.015 | 0.954 | 1.349×10^-8^ |
| Day 29 | 0.997 | 86.406 | -0.887 | 1635.889 | -0.008 | 0.969 | 9.001×10^-7^ |
| Day 30 | 0.999 | 86.163 | -0.349 | 1641.872 | -0.003 | 0.924 | 1.018×10^-8^ |
| Day 31 | 0.999 | 78.494 | -0.241 | 1641.893 | -0.002 | 0.936 | 6.324×10^-8^ |
| Day 32 | 0.999 | 79.889 | -0.148 | 1646.049 | -0.001 | 0.972 | 3.890×10^-8^ |
| Day 33 | 0.999 | 82.197 | -0.120 | 1647.900 | -0.001 | 0.984 | 3.118×10^-8^ |
| Day 34 | 0.999 | 75.306 | -0.096 | 1647.978 | -0.001 | 0.943 | 2.186×10^-8^ |

**Supplementary Table 3.** Percentage variance and cumulative variance described by the principal components attained by decomposition of sample FTIR data using principal component analysis.

| **Principal component number** | **Eigen value** | **Variance** | **Cumulative variance** |
| --- | --- | --- | --- |
|  |  | **(%)** | **(%)** |
| 1 | 3.965 | 99.13 | 99.13 |
| 2 | 0.034 | 0.86 | 99.99 |
| 3 | 5.93×10^-4^ | 0.01 | 100 |
| 4 | 1.5×10^-5^ | 0.00 | 100 |

Since the moisture content is a critical factor that determines the shelf life, the moisture contents in fresh, half and completely spoiled beetroot samples were estimated. In order to analyse and to discriminate moisture content in fresh, half and completely spoiled beetroot samples, the peak transmittance and area under the transmittance curve measured in the spectral ranges of 2614 – 4000 and 1465 – 1853 cm^-1^ were given as inputs for principal component analysis. As can be seen from loading plot (Supplementary Fig. 1), the coefficients of PC1 and PC2 for the variables $\int_{\overline{\nu}_{i}}^{\overline{\nu}_{f}} T_{p}d\overline{\nu}$ and $T_{p}$ measured in the spectral range of 2614 – 4000 cm^-1^ were estimated as 0.5, 0.5 and -0.495, -0.505 respectively. Similarly, the coefficients of PC1 and PC2 for the variables $\int_{\overline{\nu}_{i}}^{\overline{\nu}_{f}} T_{p}d\overline{\nu}$ and $T_{p}$ measured in the spectral range of 1465 – 1853 cm^-1^ were determined as 0.5, 0.5 and 0.497, 0.503 respectively. The Eigen values of the correlation matrix are given in Table 1. As can be seen from Supplementary Table 3, the first two principal components explain 99.98% of the variance and the remaining components each contribute 0.01%. Based on the analysis, the first two principal components were chosen.
